# Comprehensive Evaluation of PAXgene Fixation on Oral Cancer Tissues Using Routine Histology, Immunohistochemistry, and FTIR Microspectroscopy

**DOI:** 10.3390/biom11060889

**Published:** 2021-06-15

**Authors:** Pooja Lahiri, Suranjana Mukherjee, Biswajoy Ghosh, Debnath Das, Basudev Lahiri, Shailendra Kumar Varshney, Mousumi Pal, Ranjan Rashmi Paul, Jyotirmoy Chatterjee

**Affiliations:** 1 School of Medical Science and Technology, Indian Institute of Technology Kharagpur, Kharagpur 721302, India; suranjana.m@gmail.com (S.M.); biswajoy101@gmail.com (B.G.); debnath1985@gmail.com (D.D.); jchatterjee@smst.iitkgp.ac.in (J.C.); 2 Department of Electronics and Electrical Communication Engineering, Indian Institute of Technology Kharagpur, Kharagpur 721302, India; blahiri@ece.iitkgp.ac.in (B.L.); skvarshney@ece.iitkgp.ac.in (S.K.V.); 3 Guru Nanak Institute of Dental Sciences and Research (GNIDSR), Kolkata 700114, India; drmpal62@gmail.com; 4 Department of Oral & Dental Sciences, JIS University, Kolkata 700109, India; dr_rsspaul@yahoo.co.in

**Keywords:** PAXgene tissue fixation, formalin fixation, oral epithelial dysplasia, oral squamous cell carcinoma, immunohistochemistry, spectral histology, FTIR

## Abstract

The choice of tissue fixation is critical for preserving the morphology and biochemical information of tissues. Fragile oral tissues with lower tensile strength are challenging to process for histological applications as they are prone to processing damage, such as tissue tear, wrinkling, and tissue fall-off from slides. This leads to loss of morphological information and unnecessary delay in experimentation. In this study, we have characterized the new PAXgene tissue fixation system on oral buccal mucosal tissue of cancerous and normal pathology for routine histological and immunohistochemical applications. We aimed to minimize the processing damage of tissues and improve the quality of histological experiments. We also examined the preservation of biomolecules by PAXgene fixation using FTIR microspectroscopy. Our results demonstrate that the PAXgene-fixed tissues showed significantly less tissue fall-off from slides. Hematoxylin and Eosin staining showed comparable morphology between formalin-fixed and PAXgene-fixed tissues. Good quality and slightly superior immunostaining for cancer-associated proteins p53 and CK5/6 were observed in PAXgene-fixed tissues without antigen retrieval than formalin-fixed tissues. Further, FTIR measurements revealed superior preservation of glycogen, fatty acids, and amide III protein secondary structures in PAXgene-fixed tissues. Overall, we present the first comprehensive evaluation of the PAXgene tissue fixation system in oral tissues. This study concludes that the PAXgene tissue fixation system can be applied to oral tissues to perform diagnostic molecular pathology experiments without compromising the quality of the morphology or biochemistry of biomolecules.

## 1. Introduction

Pre-analytical handling of surgical, biopsy, or post-mortem tissue is a critical step that influences the quality of the collected biospecimen and determines the outcome of the diagnostic methods applied [1]. Ten percent neutral buffered formalin (NBF) is the fixative reagent of choice for biological tissues in the medical community for almost a century because of its excellent preservation capability [2]. The processing of tissues with NBF is relatively cheap and hassle-free and can be applied to any type of biological tissue across species. The NBF-fixed paraffin-embedded (FFPE) tissue blocks can be stored for an extended period of time, allowing the creation of disease-specific archives (tissue-biobanks) of diagnostically relevant tissues [1,2]. These tissue biobanks are often used in medical research to investigate and teach the pathogenesis of a variety of diseases [3,4]. 

In recent years, molecular diagnostic pathology has widened its approaches to assess disease progression. Histo-morphological assessment with alterations in genetic material and functional biochemistry of disease-associated biomolecules is fast becoming the norm for a comprehensive diagnosis [1,2,4,5]. Therefore, enormous efforts are being made by the scientific and clinical community to isolate and preserve a good quality of nucleic acids and proteins from FFPE tissue blocks for molecular diagnostic assays. However, the success rates of these efforts are marginalized by the harsher treatments required to break the nucleic acid and protein crosslinks formed by NBF fixation, rendering the isolated samples not suitable for molecular analysis [6,7]. Currently, snap freezing of tissues and storage at low temperatures in liquid nitrogen is considered appropriate for molecular analysis [3,8]. Nevertheless, liquid nitrogen preservation comes with the possibility of intracellular ice build-up, leading to defects in the morphology of tissue [9]. Hence, the choice of tissue fixative should be made carefully when new biomarker studies on disease progression are initiated to ensure that the antigenicity of the epitopes is not compromised, and the chemistry of crucial biomolecules is preserved. Newer methods of fixation and preservation of tissues are always wanted in clinical diagnostics.

In this study, we have characterized the new PAXgene tissue fixation and stabilization reagent on oral buccal mucosal biopsied tissues for routine histological and immunohistochemical applications [8]. Oral buccal mucosal tissue has the lowest tensile strength among other intraoral sites, such as the gingiva and hard palate [10]. Hence, it is not surprising that oral buccal mucosal tissue suffers from histological processing errors, such as tissue fall-off from the slides and wrinkling. We observed continuing difficulty in handling FFPE oral buccal mucosal tissues for immunohistochemical studies and routine histological staining, leading to delays in experimental results, non-reproducibility of data, and loss of useful molecular information. Therefore, we aimed to optimize the oral tissue fixation and histological staining procedures with PAXgene tissue fixation reagents. According to the manufacturer (QIAGEN), the PAXgene tissue fixation system is a non-toxic method of tissue fixation consisting of mixtures of alcohols, acetic acid, and soluble organic compounds [8]. It is claimed that the PAXgene fixation reagent rapidly preserves the morphology of the biomolecules [11]. The fixation is followed by adding a stabilization reagent consisting of a mixture of alcohol that enhances the stability of the bio-specimens for the transport of samples and long-term preservation. We have further investigated the preservation of biomolecules by PAXgene fixation by FTIR microspectroscopy. The FTIR spectrum provides information on the overall biochemical status of major biomolecules, i.e., nucleic acids, lipids, and proteins in tissue. The variations in biomolecule chemistry in response to external factors (in this study—tissue fixation methods) can be calculated by assessing the changes in infrared spectra [12]. In this study, we thoroughly evaluated the PAXgene fixation with conventional formalin fixation with an aim to minimize the processing damage of tissues and improve the quality of the histopathological diagnosis.

## 2. Materials and Methods

### 2.1. Collection of Tissue and Fixation

Patients showing clinical symptoms of oral pre-cancers and oral squamous cell carcinoma (OSCC) were chosen for the biopsy procedure. All biopsies were performed at Guru Nanak Institute of Dental Sciences and Research (GNIDSR). A wedge biopsy was performed to collect representative tissues from pre-cancerous or cancerous areas in the buccal mucosa of selected patients. Another tissue biopsy was performed on the adjacent buccal mucosa to the pre-cancerous or cancerous site, clinically representing a normal architecture or normal oral mucosa (NOM). Further, all types of collected biopsied tissues were divided into two equal parts. One part of the tissue was added to freshly prepared 10% NBF (tissue_F) and the other part of the tissue was added into PAXgene tissue fix (QIAGEN) reagent (tissue_P). After 48 h of fixation, fresh 10% NBF formalin was added to the tissue_F, and the PAXgene tissue stabilizer reagent was added to the tissue_P followed by gentle agitation (250 rpm) for 72 h. Post stabilization of tissue_P, both tissue_F and tissue_P were processed for paraffin embedding according to the established protocol reported elsewhere [3,8,13]. In this article, formalin-fixed paraffin-embedded tissues and PAXgene-fixed paraffin-embedded tissues will be referred to as FFPE and PFPE, respectively. The sample size for the different experimental set-ups is mentioned whenever required. This study was performed with the informed consent of all individuals and approved by the institutional ethics committee (GNIDSR/IEC/07/16).

### 2.2. Hematoxylin and Eosin Staining

Histological assessment of FFPE and PFPE tissues in terms of preservation of morphology (overall morphology and nuclear, cytoplasmic, and membrane details) was performed on 4 µm tissue sections using hematoxylin and eosin (H&E) staining and verified by at least three certified oral pathologists from GNIDSR, Kolkata. The tissue sections were mounted on AUTOFROST charged adhesion microscopic slides (#20190710; Cancer Diagnostics Inc., , Durham, NC, USA.

For H&E staining, FFPE and PFPE tissue sections were deparaffinized at 65 °C for 30 min, followed by xylene treatment for 20 min. The rehydration of the tissue sections was performed with decreasing gradients of ethanol (100%, 90%, 70%, 50%, and MilliQ water). Tissue slides were stained by immersing in Mayer’s Hematoxylin solution (#51275, Sigma Aldrich, Bangalore, India) for 1 min, followed by a tap water wash for 5 min. Counterstaining with Eosin Y stain (#E4009, Sigma Aldrich) was performed for 20 s. Dehydration with an increasing gradient of ethanol was immediately done after counterstaining. The slides were cleared with xylene for 5 min and mounted with DPX. The integrated density value of the hematoxylin and eosin stain for PFPE-fixed and FFPE-fixed NOM, dysplasia, and OSCC tissues were calculated using the ImageJ (NIH, Bethesda, MD, USA) color deconvolution tool (HE filter,). Statistical analysis was performed using GraphPad Prism software (GraphPad, San Diego, CA, USA).

### 2.3. Immunohistochemistry

Immunohistochemical staining for epithelial cytokeratin CK5/6 (#ab5312 and # ab18586, Abcam, Cambridge, UK) and tumor suppressor protein p53 (#ab131442, Abcam, Cambridge, UK) was performed on 4 µm tissue sections prepared from FFPE and PFPE blocks according to manufacturer’s instructions (#K8023, DAKO, Santa Clara, CA, USA), with modifications in the antigen retrieval methodology (Table S2). Both FFPE and PFPE tissue slides were deparaffinized at 65 °C for 30 min, followed by xylene treatment for 30 min. The tissue sections were re-dehydrated by immersing the slides consecutively in 100% ethanol, 90% ethanol, 70% ethanol, and 50% ethanol for 2 min each. All the tissue slides were given a brief MilliQ water wash prior to antigen retrieval. As oral biopsied tissues are sensitive to tissue fall-off during microwave/pressure cooker procedures, antigen retrieval for FFPE tissue was performed by immersing the slides with tissue sections in TRIS EDTA pH 9 buffers at 65 °C for 30 min to minimize tissue disruption (Table S2). Identical antigen retrieval conditions were maintained for PFPE tissue. In addition, PFPE tissue sections were also processed without heat-induced antigen retrieval. After antigen retrieval (if performed), tissue slides were brought back to room temperature prior to the antibody steps. Primary antibody dilution was prepared according to the manufacturer’s instruction (p53:1:500; CK5/6, 1:500) in 1% BSA + TBS. The tissue slides were incubated with primary antibody overnight. The next day, ready to use secondary antibody (EnVision™ FLEX/HRP; DAKO) was added to the TBS-washed tissue sections and incubated for 1 h. Following secondary antibody application, sections were properly washed in TBS, and DAB chromogen was added for the color reaction. The appropriate time was noted for the color development (p53; 2.5 min and CK5/6; 1 min) and was maintained for all the experiments performed. All the tissue slides were counterstained with Mayer’s Hematoxylin, dehydrated using ethanol, and mounted using DPX (#06522, Merck, Bangalore, India) (Method S1). The integrated density value of each immuno-stain (p53 and CK5/6) for PFPE-fixed and FFPE-fixed NOM, dysplasia, and OSCC tissues were calculated using the ImageJ (NIH, Bethesda, MD., USA) color deconvolution tool (H DAB filter). Statistical analysis was performed using GraphPad Prism software (GraphPad Prism, San Diego, CA, USA).

### 2.4. Microscopy

H&E and immunohistochemistry images were captured using a brightfield microscope (Leica Microsystems, Wetzlar, Germany) at different magnifications (10×, 20×, and 40×). Scale bars were added using Image J. All the H&E images and IHC images were assembled as figures using ImageJ, Adobe Illustrator CC (Adobe, San Jose, CA, USA), or MS PowerPoint software.

### 2.5. FTIR Microspectroscopy

Tissue sections of 4 µm in thickness were prepared from both FFPE and PFPE blocks for FTIR microspectroscopy. Of note, 4 µm sections for H&E staining and immunohistochemistry were prepared simultaneously and are considered parallel sections for selected experiments. The 4 µm tissue sections on AUTOFROST slides were baked at 60 °C for 30 min, followed by two changes of xylene for deparaffinization. Immediately, the tissue sections were dehydrated by immersing the slides in 100% ethanol for 10 min. The tissue sections were then allowed to air dry for 30 min at room temperature. The tissue sections were analyzed under a LUMOS-II FTIR microspectroscope (Bruker, Billerica, MA, USA) with a liquid nitrogen-cooled mercury-cadmium-telluride 64 × 64-pixel focal plane array (FPA) detector within the same day post dehydration. Histologically diagnosed FFPE-NOM, PFPE-NOM, and FFPE-OSCC, PFPE OSCC were used for measurement of the spectra. Spectroscopic imaging data were acquired in reflection mode. Background spectra were collected from a region free of tissue of the FTIR-reflective slide. Spectral data were recorded using a mirror speed of 1 cm^−1^, a spectral resolution of 4 cm^−1^, and using an acquisition time of 6 s (64 scans) per measurement. Twenty tissues from each group were used for spectral data acquisition. In total, 200 spectral measurements were recorded for FFPE-NOM (N^spectra^ = 100) and PFPE-NOM (N^spectra^ = 100), and 300 spectral measurements were recorded for FFPE-OSCC (N^spectra^ = 150) and PFPE-OSCC tissue (N^spectra^ = 150). Pathologically similar regions of interest exhibiting a normal oral morphology of the epithelium were chosen for spectral measurements from the NOM group of tissues. Moreover, epithelial islands from well-differentiated squamous cell carcinoma tissues were chosen for spectral measurements from the OSCC group of tissues. Average spectra were calculated and smoothened for 25 smoothening points (Savitzky Golay algorithm) for each tissue group only after ascertaining no significant variation between the average spectra and individual spectra taken from each region of interest. No other post-processing of spectra was performed. The band position for each bio-component was calculated using the peak analyzer algorithm of Origin Pro software (Northampton, MA, USA). 

**Figure 1:**
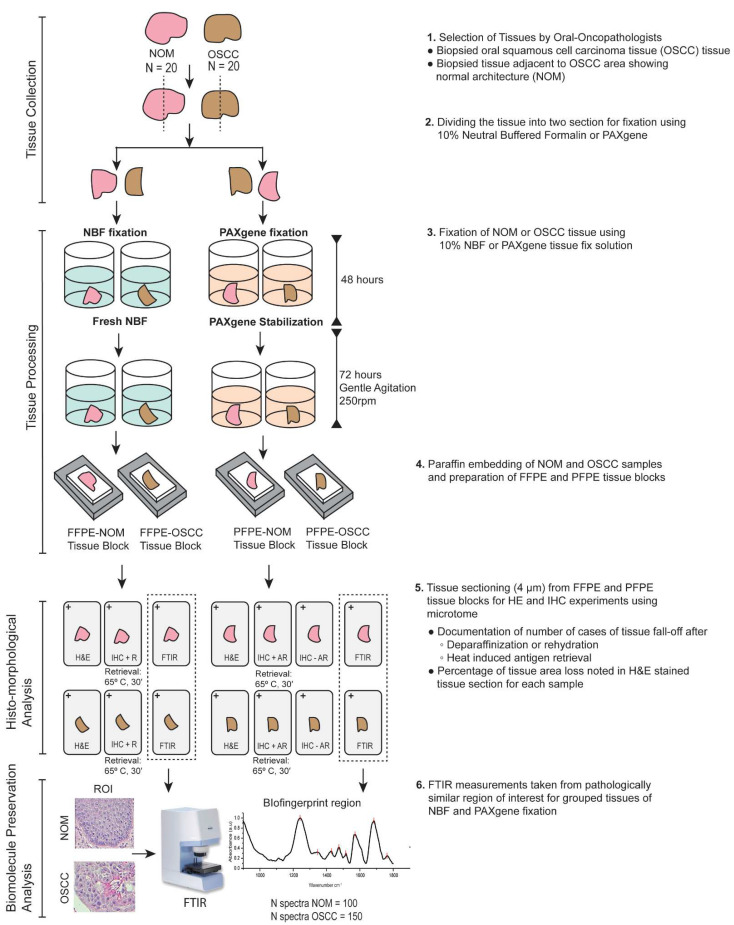
Sample collection and experimental setup of the study. Abbreviations: H&E, hematoxylin and eosin; IHC, immunohistochemistry; R, retrieval; FTIR, Fourier-transform infrared; AR, antigen retrieval.

Sample collection and the experimental set-ups are depicted in Figure 1.

## 3. Results

### 3.1. Histological Comparison of FFPE and PFPE Tissues Using H&E Staining 

FFPE oral buccal mucosal tissues with a normal architecture (NOM) are challenging to process for histological staining. We frequently experienced tissue tear, wrinkling, and fall-off of the tissue-section from slides, either after deparaffinization at 65 °C or after rehydration with a decreasing gradient of alcohol. We maintained a log of all the FFPE samples received between 2019 and 2020 from the GNIDSR tissue bank and documented the cases of tissue fall-off from slides. Around 28.6% of the FFPE-NOM cases could not be processed further for histological staining after the rehydration step due to the fall-off of tissue sections from the slides (Table 1). However, only 5.5% of FFPE-OSCC cases suffered tissue section fall-off from slides (Table 1). 

To minimize the delay in experimentation due to the loss of tissue sections during the processing of FFPE samples, we used PAXgene as an alternative tissue fixation method for oral buccal mucosal tissue. We investigated whether the change in fixation method of oral tissues reduces the problem of tissue section fall-off, tissue tear, or wrinkling. For this purpose, we selected a total of 40 fresh biopsied tissues (NOM: *n* = 20; OSCC: *n* = 20), divided them into two parts, and fixed them in freshly prepared 10% NBF or PAXgene tissue fixative solution (Figure 1). Tissue sections of 4 µm in thickness were prepared from FFPE and PFPE tissue blocks and processed for H&E staining. We noted seven cases of tissue section fall-off for the FFPE-NOM (*n* = 20) tissue sample compared to three cases for the PFPE-NOM tissue samples (*n* = 20) (Table 2). Further analysis on H&E-stained sections showed the percentage of loss of epithelial and sub-epithelial tissue area due to tissue tear or wrinkling, which was more prominent in FFPE-NOM samples than PFPE-NOM samples (Table S1). Moreover, no cases of tissue section fall-off were noted for the PFPE-OSCC tissue samples (*n* = 20), and only one case was noted for the FFPE-OSCC tissue samples (Table 2). 

**Figure 2:**
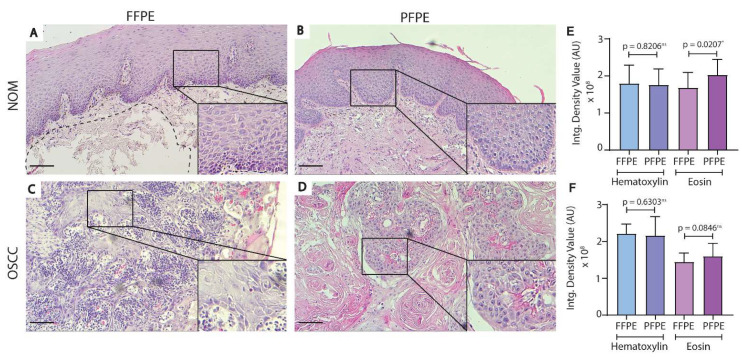
Morphological comparisons of PFPE and FFPE tissues. Hematoxylin and eosin staining was performed on FFPE NOM and OSCC tissues (**A**,**C**) and PFPE NOM and OSCC tissues (**B**,**D**). PFPE tissues were more eosinophilic than FFPE tissues (**A**,**B**)—corresponding integrated density value plot (**E**)—and (**C**,**D**)—corresponding integrated density value plot (**F**). Morphology of the epithelium layers and sub-epithelium region was well-preserved in PFPE-NOM tissue (**B**) and was comparable to FFPE-NOM tissue (**A**). Squamous cell carcinoma was well-represented in both PFPE (**C**) and FFPE tissue (**D**). High magnification insets for each panel (**A**–**D**) shows the morphology of the epithelial cells in NOM (**A**,**B**) and OSCC (**C**,**D**). An example of loss of tissue area (dotted lines) due to sample processing was shown in FFPE-NOM tissue (**A**). Magnification = 10×; Scale bar = 50 µm. *p* < 0.05 * (student’s *t*-test). Abbreviations: PFPE, PAXgene-fixed paraffin embedded; FFPE, formalin-fixed paraffin embedded; NOM, normal oral mucosa; OSCC, oral squamous cell carcinoma; Intg., integrated.

In terms of overall histology, the morphology of the H&E-stained sections of FFPE-NOM and PFPE-NOM or FFPE-OSCC and PFPE-OSCC was similar (Figure 2). A slightly enhanced eosinophilic reaction was observed in both the epithelium and sub-epithelium regions of PFPE-NOM and PFPE-OSCC tissues (Figure 2A–F). This enhanced the cytoplasmic and nuclear details and did not hamper the histological evaluation of the disease morphology (Figure 2B,D).

### 3.2. PFPE Tissues Do Not Require Heat-Induced Antigen Retrieval for Immunohistochemistry

After we observed comparatively fewer tissue section fall-off cases for PFPE tissue samples, we tested the PFPE-NOM and PFPE-OSCC tissue samples for immunohistochemical applications. In particular, we assessed the expression of tumor suppressor protein p53 and cytoskeletal cytokeratins (CK) 5 and 6 by performing immunohistochemistry with respective antibodies on FFPE and PFPE tissue sections. Our laboratory experienced an increase in tissue fall-off cases from slides after the heat-induced antigen retrieval step for FFPE tissues (Table 3). We tried various heating and pressure conditions for optimum antigen retrieval with FFPE-NOM tissues (*n* = 20) (Table S2) and to minimize tissue fall-off and damage (Table 4). The optimum antigen-retrieval was observed after heating the samples at 65 °C for 30 min with tris-citrate buffer, pH 6, in a water bath (Table S2). 

**Figure 3:**
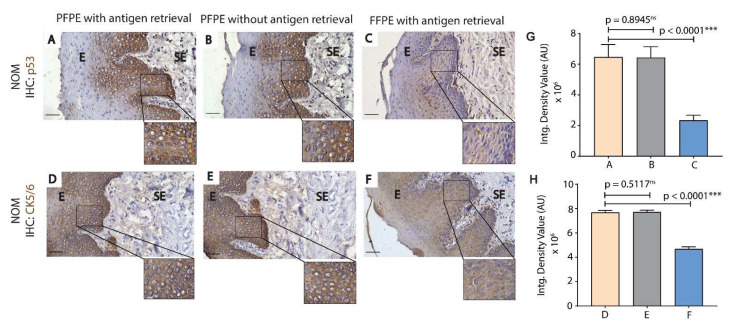
Heat-induced antigen retrieval is not essential for PFPE-NOM tissues. Immunohistochemistry with antibodies against p53 (**A**–**C**; upper panel) and CK5/6 (**D**,**E**; lower panel) was performed on PFPE-NOM (**A**,**B**,**D**,**E**) and FFPE-NOM (**C**,**F**) tissue sections. Parallel tissue sections of PFPE-NOM samples were subjected to primary antibody (p53 or CK5/6) incubation with a prior antigen retrieval step (**A**,**D**) and without an antigen retrieval step (**B**,**E**). Antigen retrieval was performed on tissue sections of FFPE-NOM samples (**C**,**F**) before antibody (p53 or CK5/6) incubation (**C**,**F**). p53 immuno-staining was observed in basal and suprabasal layers of the epithelium in PFPE-NOM tissues with (**A**) or without antigen retrieval (**B**). However, the intensity of p53 immuno-staining was markedly less in FFPE-NOM tissues (corresponding integrated density value plot (**G**) for **A**–**C**) with no or noticeably light staining of p53 in basal cells. Similarly, CK5/6 immuno-staining was observed homogenously throughout the epithelium in PFPE-NOM tissues with (**D**) and without antigen retrieval (**E**). In FFPE-NOM tissue, CK5/6 immuno-staining was observed through-out the epithelium, but with markedly less intensity than PFPE-NOM tissues (corresponding integrated density value plot (**H**) for **D**–**F**). The incubation time for DAB chromogen was 2.5 min for p53 antibody and 1 min for CK5/6 antibody. Magnification = 20×; Scale bar = 25 µm. *p* < 0.0001 *** (student *t*-test). Abbreviations: E, epithelium, SE, sub-epithelium; AR, antigen retrieval; FFPE, formalin-fixed paraffin embedded; PFPE, PAXgene-fixed paraffin embedded; NOM, normal oral mucosa; CK, cytokeratin; Intg, integrated.

Unlike formalin, the PAXgene tissue fixation solution does not form methylene crosslinks in tissues [8]. Hence, a protocol deviation was introduced for PFPE tissues where a parallel section was not subjected to antigen retrieval (Figure 3B,E). We observed cytoplasmic and nuclear expression of p53 in the basal and suprabasal layer of the epithelium and no difference in p53 immuno-expression between PFPE-NOM tissue sections with and without antigen retrieval (Figure 3A,B,G). However, the intensity of the p53 immuno-expression was significantly less in the suprabasal layer, and no or noticeably light staining was observed in FFPE-NOM tissue when compared to PFPE-NOM tissues (Figure 3C vs. Figure 3A,B,G) in replicated experiments even with an increase in the timing of DAB staining to 2.5 min (data not shown). We observed a homogenous cytoplasmic expression of CK5/6 in the epithelium of PFPE-NOM and FFPE-NOM tissue; however, similar to p53, the intensity of expression of CK5/6 was significantly less (Figure 3H) in the epithelium layers of FFPE-NOM tissue (Figure 3F vs. Figure 3D,E). Furthermore, no difference in CK5/6 immuno-expression was observed between PFPE-NOM tissues with or without antigen retrieval (Figure 3D,E,H).

**Figure 4:**
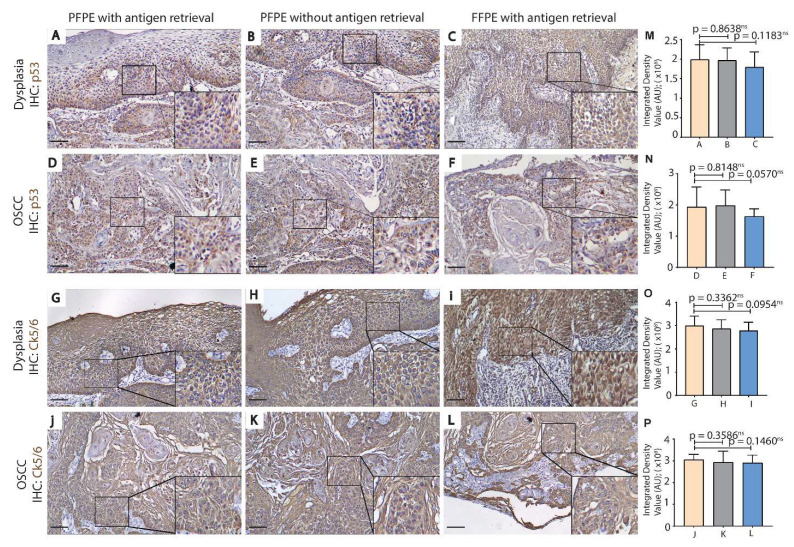
Heat-induced antigen retrieval is not essential for PFPE-Dysplasia and PFPE-OSCC tissues. Immunohistochemistry with antibodies against p53 (**A**–**F**) and CK5/6 (**G**–**L**) was performed on PFPE-Dysplasia (**A**,**B**,**G**,**H**), PFPE-OSCC (**D**,**E**,**J**,**K**), FFPE-Dysplasia (**C**,**I**), and FFPE-OSCC (**F**,**L**) tissues. Parallel tissue sections of PFPE-Dysplasia and PFPE-OSCC tissue were subjected to primary antibody (p53 or CK5/6) incubation with a prior antigen retrieval step (**A**,**D**,**G**,**J**) and without an antigen retrieval step (**B**,**E**,**H**,**K**). Antigen retrieval was performed on tissue sections of FFPE-Dysplasia (**C**,**I**) and FFPE-OSCC (**F**,**L**) before antibody (p53 or CK5/6) incubation (**C**,**F**). A similar intensity of the p53 immuno-staining was observed in basal and suprabasal layers of the epithelium in PFPE-Dysplasia tissues (corresponding integrated density value plot (**M**) for **A**–**C**) with (**A**) or without antigen retrieval (**B**), and for FFPE-Dysplasia (**C**). CK5/6 immuno-staining was observed homogenously (corresponding integrated density value plot (**O**) for **G**–**I**) through-out the epithelium in PFPE-Dysplasia tissues with (**G**) or without antigen retrieval (**H**), and for FFPE-Dysplasia (**I**). In PFPE-OSCC tissues, p53 immuno-expression (**D**,**E**) was found in epithelial islands and CK5/6 (**J**,**K**) immuno-expression was found in epithelial islands and keratin pearls, and the immunostaining and localization of both proteins were comparable to FFPE-OSCC tissues (**C**,**F**,**I**,**K**) (corresponding integrated density value plot (**N**,**P**) for **G**–**I** and **J**–**L**, respectively). The incubation time for DAB chromogen was 2.5 min for the p53 antibody and 1 min for the CK5/6 antibody. Magnification = 10×; Scale bar = 50 µm. *p* < 0.0001 ***; *p* < 0.001 **; *p* < 0.05 * (student *t*-test). Abbreviations: FFPE, formalin-fixed paraffin embedded; PFPE, PAXgene-fixed paraffin embedded; OSCC, oral squamous cell carcinoma; CK, cytokeratin; Intg., integrated.

Next, we analyzed the expression profile of p53 and CK5/6 in cases of dysplasia and OSCC. A higher expression of p53 and CK5/6 was observed in dysplasia and OSCC tissues than NOM tissues (Figure 4 vs. Figure 3). No significant differences in the expression pattern of p53 and CK5/6 in dysplasia or OSCC were observed between PFPE tissues with antigen retrieval (Figure 4A,D,G,J); PFPE tissues without antigen retrieval (Figure 4B,E,H,K); and FFPE tissues (Figure 4C,F,I,L). However, the density value plot for p53 and CK5/6 showed a trend of a decrease in immuno-staining for both proteins in FFPE tissues compared to PFPE tissues (Figure 4M–P). Furthermore, p53 was observed in the cytoplasm and nucleus of the basal and suprabasal cells in dysplasia (Figure 4A–C) and epithelial islands in OSCC (Figure 4D–F). Likewise, CK5/6 expression was cytoplasmic and spread homogeneously throughout the epithelium layers in dysplasia (Figure 4G–I). In OSCC, cytoplasmic expression of CK5/6 was homogenously expressed in the cytoplasm of epithelial islands and the keratin pearls (Figure 4J–L). 

### 3.3. FTIR Microspectroscopy Revealed Comparable Preservation of Bio-Components between FFPE Tissues and PFPE Tissues

**Figure 5:**
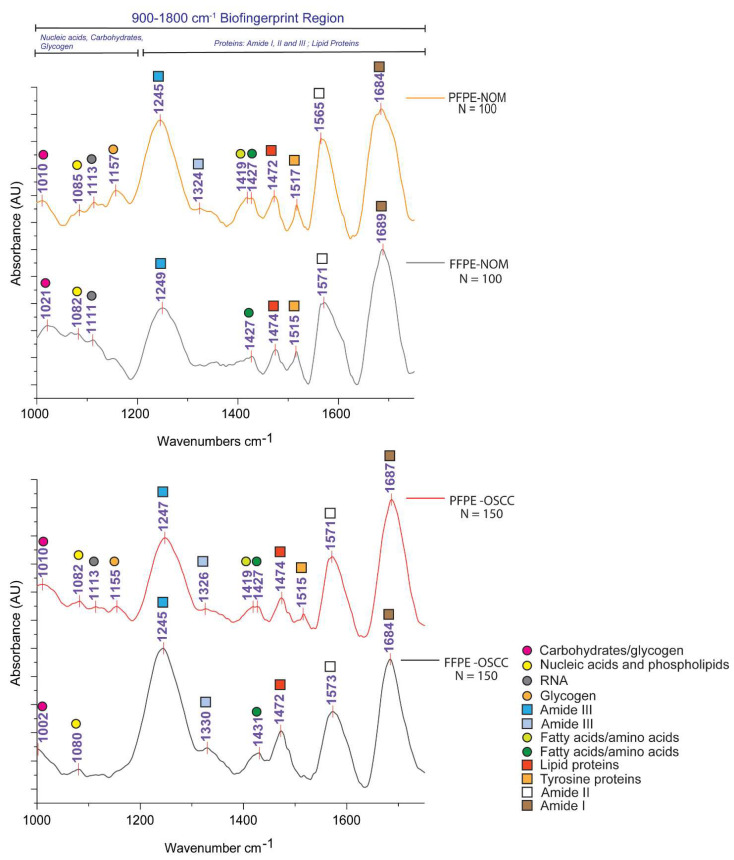
Average FTIR spectra from PFPE-NOM, FFPE-NOM, PFPE-OSCC, and FFPE OSCC tissues depicting the biofingerprint region 900 cm^−1^–1700 cm^−1^. N^spectra^ PFPE-NOM = 100; N^spectra^ FFPE-NOM = 100; N^spectra^ PFPE-OSCC = 150; and N^spectra^ FFPE-OSCC = 150. Abbreviations: FFPE, formalin-fixed paraffin embedded; PFPE, PAXgene-fixed paraffin embedded; OSCC, oral squamous cell carcinoma; NOM, normal oral mucosa.

FTIR microspectroscopy is an emerging technique in the field of ex-vivo diagnostics. FTIR spectra provide an overall estimation of the biochemical content inside tissues and can be analyzed to differentiate between disease states [12]. However, it has become apparent that methods for tissue preservation can affect or change the biochemical spectra and may lead to a false interpretation of the data [12,14,15]. We have assessed the bio-fingerprint region (900–1700 cm^−1^) to compare the effects of formalin and PAXgene tissue fixation on the spectral signatures of various bio-components in NOM and OSCC. Figure 5 represents the average spectra from each group of samples analyzed, and Table 5 depicts the band assignment [12,15] of the analyzed spectral peaks (N^spectra^ = 100, PFPE-NOM and FFPE-NOM; N^spectra^ = 150, PFPE-OSCC and FFPE-OSCC) for the bio-fingerprint region of each tissue sample group. 

The spectral region 1000–1200 cm^−1^ corresponds to absorption bands from several bio-molecules: carbohydrates, nucleic acids, and phospholipids. PAXgene tissue fixation exclusively preserved the C–O stretch vibrations corresponding to glycogen at 1157 cm^−1^ in PFPE-NOM and 1155 cm^−1^ in PFPE-OSCC. A low-frequency band assigned to glycogen/carbohydrates was centered around ~1010 cm^−1^ in the PAXgene- and NBF-fixed tissues. The ~1115 cm^−1^ band assigned to RNA (C–O stretch of the ribose ring vibrations) was found in both PAXgene- and NBF-fixed tissue (Table 5, Figure 5). 

To understand the preservation of proteins by PAXgene fixation, we analyzed the spectral region between 1200 and 1700 cm^−1^. We observed the primary amide III band centered around ~1245 cm^−1^ for the PAXgene- and NBF-fixed tissues. Additional amide III bands were observed at 1324 cm^−1^ in PFPE-NOM tissues and at 1326 cm^−1^ in PFPE OSCC tissues. The 1324 cm^−1^ band was absent in FFPE-NOM tissues; however, a 1330 cm^−1^ band was found in FFPE OSCC tissues (Table 5, Figure 5). These bands may correspond to the secondary structure of proteins corresponding to the amide III region [14]. The amide II band and amide I band were centered around ~1571 cm^−1^ and ~1684 cm^−1^, respectively, for all tissue groups. 

A high-frequency band associated with COO^-^ symmetric stretch vibrations centered around 1427 cm^−1^ assigned to fatty acids/amino acids was preserved in all tissue groups. Interestingly, the low-frequency band attributed to fatty acids centered at 1419 cm^−1^ was found exclusively in PAXgene-fixed NOM and OSCC tissues. Further, the band corresponding to CH_3_ and CH_2_ deformations assigned to lipids was found at ~1472 cm^−1^ in all tissue groups.

## 4. Discussion

Oral buccal mucosal tissues have low tensile strength when compared to other intraoral sites such as the gingiva and hard palate [10]. Thus, it is not surprising that processing of oral buccal mucosal tissue for histological experiments is a tedious process and suffers from processing errors such as tissue fall-off from slides, wrinkling, and tissue tear. This leads to unnecessary experimental delay and loss of critical morphological or molecular information. We have successfully used the formalin-free PAXgene tissue fixation system for oral buccal mucosal tissue of normal and cancer morphology without compromising the quality of the histopathological analysis (Figure 2). In line with previous observations [5,8,11], a slightly high eosinophilic reaction was observed in H&E-stained PFPE tissues (Figure 2E,F), which did not interfere with the histopathological analysis. According to the participating oral pathologists in this study, architectural alterations in the surface epithelium and individual cell alterations [16] were detectable and comparable to formalin fixation in both H&E-stained PFPE-NOM and PFPE-OSCC tissues. 

Immunohistochemistry, by far, is the most routinely used method to analyze specific cancer biomarkers and, to an extent, has advanced our knowledge on oral cancer progression [17,18]. The heat-induced antigen retrieval step is a critical step in immunohistochemistry; however, direct boiling of FFPE-NOM and FFPE-OSCC tissue in buffers results in the damage of tissues or fall-off of tissues from the slide. Several factors have been identified to contribute to the problem of tissue fall-off from slides, such as inadequate fixation, uneven sectioning and drying, and poor quality of adherent and uncleaned slides [19]. Even after taking care of the aforementioned factors, we observed recurrent tissue fall-off from slides of FFPE-NOM and FFPE-OSCC samples after boiling (Table 3 and Table 4). PAXgene-fixed tissues show a slight reduction in tissue-fall from slides after boiling (Table 4). As PAXgene tissue fixation systems do not form crosslinks with the tissues, we removed the antigen-retrieval step for the PFPE-NOM and PFPE-OSCC samples and successfully optimized the immunohistochemistry protocol for oral cancer biomarkers p53 and CK5/6. Skipping the antigen retrieval step significantly reduced the problem of tissue fall-off from slides (Table S3). Although antigen retrieval by boiling is an excellent way to gain the immunoreactivity of antibodies, it also damages the morphology of the tissue to some extent, which may hinder diagnosis. Further, the consensus based on previous observations on FFPE tissue is that every antigen requires a “tailor-made” retrieval condition, which is highly prone to technical errors, leading to irreproducibility of the data [20]. Our experiments showed improved quality of immuno-staining of both p53 and CK5/6 in basal and suprabasal epithelial layers for PFPE-NOM tissues (with or without antigen retrieval) over FFPE-NOM tissues (with antigen retrieval) (Figure 4). This is an important observation as any redundancies in the control group may lead to incorrect interpretation of the expression of cancer-associated proteins (in this case—p53 and CK5/6), false diagnosis, and incorrect statistics in comparative datasets. For dysplasia and OSCC samples, the immuno-staining in PFPE tissues was comparable to FFPE tissues as per the oral-pathologists’ interpretation (Figure 4). However, a semi-quantitative analysis on the density value of the immuno-stains shows less expression of p53 and CK5/6 in FFPE-tissues (Figure 3 and Figure 4). Recently, concerns have been raised regarding the irreproducibility of the immunohistochemical data for studying prognostic features in OSCC [21,22]. The 8th edition of the American Joint Committee on Cancer (AJCC) staging has reinforced focus on histo-morphological prognostic features, such as tumor stroma ratio, depth of invasion, and extranodal extension, to study the prognosis of OSCC [22]. From our experiments, it can be inferred that PAXgene tissue fixation of oral mucosa tissues can be successfully implemented in histo-morphological and molecular pathology studies, with enhanced staining characteristics and minimal risk of irreproducibility.

Next, we investigated the preservation of biochemical content of oral samples using FTIR microspectroscopy. We measured and analyzed spectra from pathologically similar region of interests from grouped tissues of NOM and OSCC fixed with either NBF or the PAXgene tissue fixation system (Figure 1). PAXgene fixation, and not NBF fixation, showed preservation of the C–O vibrations attributable to glycogen (Figure 5 and Table 5). Previous histological studies have shown a higher glycogen expression in oral dysplasia when compared to normal, and it is an indicator of cancer progression [23,24]. Structural alterations in nucleic acids, and increased transcriptional activity pertaining to increase in RNA, was previously reported in various studies on OSCC [17,24]. The RNA band corresponding to the C–O stretch of the ribose ring was preserved in both PAXgene and NBF-fixed tissues (Figure 5 and Table 5). Further, prior studies have shown PAXgene fixation is superior in preserving RNA integrity, particularly in low-yield samples for molecular experiments [3,5,25]. It also was reported that the PAXgene-fixed RNA has better RT-PCR efficiency than NBF fixation and was comparable to snap-frozen samples, irrespective of the length of the mRNA, in a wide variety of cancerous tissues, including squamous cell carcinoma [5,25,26]. Further in-depth comparative studies are warranted in oral tissue to understand the effect of PAXgene fixation on RNA integrity and downstream analysis such as RT-PCR and RNA sequencing.

In terms of the preservation of proteins, all three amide bands (amide I, amide II, and amide III) were preserved in both PAXgene- and NBF-fixed tissues. We also observed an additional band for amide III centered around ~1324 cm^−1^ in both PFPE-NOM and PFPE OSCC tissues (Table 5). These bands correspond to the alpha-helical secondary structure of proteins [14]. The amide I region is generally used for studying the secondary structure modifications in proteins, whereas the amide III region is comparatively neglected due to the low signal. However, it was previously described that the secondary structures of proteins have more resolved differences in their amide III spectra [14,27]. Recent studies have indicated that the secondary structure modifications are a direct measure of the gain-of-function mutations of the amino acids of proteins leading to cancer progression [28,29]. Furthermore, alterations in secondary structure of proteins, to an extent, modulates the phosphorylation of proteins [30]. Recently, a MS-based approach showed 62 unique phosphorylated sites in the tumor epithelium of OSCC [31]. In this regard, prior studies have shown enhanced preservation of phosphorylated proteins in PAXgene-fixed tissues than formalin-fixed tissues [26,32]. Further studies on secondary structure alterations in PAXgene-fixed NOM and OSCC tissues may provide new insights into the protein biomarkers of oral cancer progression.

## 5. Conclusions

To the best of our knowledge, this is the first time that PAXgene and NBF fixation of oral tissues were simultaneously compared in terms of morphology (histology, immunohistochemistry) and the sample biochemical variations arising from different methods of fixation (FTIR). Our study demonstrates that for fragile tissues such as oral buccal mucosa, which are prone to processing and handling errors, PAXgene tissue fixation systems can be implemented for routine histological and immunohistochemistry experiments without compromising on the quality of the morphological diagnosis. PAXgene fixation also ensured the preservation of several important biocomponents (nucleic acids, lipids, and proteins) as revealed by FTIR microspectroscopy. This information will save time in the clinics while collecting and processing the oral tissue for molecular diagnostic purposes as a second fixative for the preservation of nucleic acids may not be required. As medical decision-making moves towards personalized diagnostics by obtaining a broader spectrum of information from the same tissue, the PAXgene tissue fixation system may help improve the efficiency of diagnosis.

## Figures and Tables

**Table 1:** Documentation of cases of tissue section fall-off from slides during sample processing steps for histochemical staining experiments for a period of 1 year (2019–2020) for FFPE tissues from the tissue bank of GNIDSR.

	Total Number of FFPE Tissue ^a^	Cases of Tissue Fall-Off *	Percentage
Number of NOM samples	35	10	28.57
Number of OSCC samples	73	8	5.48

^a^ Number of tissue samples (FFPE) received during 2019–2020 from GNIDSR, Kolkata. * Number of cases of tissue fall-off from slides after either deparaffinization or rehydration of the tissue section with a decreasing gradient of alcohol. Abbreviations: NOM, normal oral mucosa; OSCC, oral squamous cell-carcinoma; FFPE, formalin-fixed paraffin embedded; PFPE, PAXgene-fixed paraffin embedded.

**Table 2:** Documentation of cases of tissue section fall-off from slides during the sample processing steps for histochemical staining experiments for matched FFPE and PFPE tissue biopsies.

	Total Number of FFPE Tissues ^a^	Cases of Tissue Fall-Off *	Percentage	Total Number of PFPE Tissues ^a^	Cases of Tissue Fall-Off *	Percentage
Number of NOM samples	20	7	35.00	20	3	15.00
Number of OSCC samples	20	1	5.00	20	0	0.00

^a^ Number of tissue samples (FFPE or PFPE) received from GNIDSR, Kolkata, for the comparative study. * Number of cases of tissue fall-off from slides after either deparaffinization or rehydration of the tissue section with a decreasing gradient of alcohol. Abbreviations: NOM, normal oral mucosa; OSCC, oral squamous cell-carcinoma; FFPE, formalin-fixed paraffin-embedded; PFPE, PAXgene-fixed paraffin-embedded.

**Table 3:** Documentation of cases of tissue section fall-off from slides during sample processing steps for immuno-histochemical staining experiments for a period of 1 year (2019–2020) for FFPE tissues.

	Total Number of FFPE Tissues *	Cases of Tissue Fall-Off after D or R Steps	Cases of Tissue Fall-Off after AR	Total Cases of Tissue Fall-Off	Number of Cases Left for IHC Expts.
Number of NOM samples	35	12	18	30	5
Number of OSCC samples	73	3	43	46	30

* Number of tissue samples (FFPE) received during 2019–2020 from GNIDSR, Kolkata. Abbreviations: D, deparaffinization; R, rehydration; NOM, normal oral mucosa; OSCC, oral squamous cell-carcinoma; FFPE, formalin-fixed paraffin embedded; AR, antigen retrieval; IHC, immunohistochemistry.

**Table 4:** Documentation of cases of tissue section fall-off from slides during sample processing steps for immune-histochemical staining experiments for matched PFPE and FFPE tissue biopsies.

	Number of NOM Samples	Number of OSCC Samples
**Total number of FFPE tissues**	20	20
Cases of tissue fall-off after D or R steps	6	2
Cases of tissue fall-off after AR	12	9
Total cases of tissue fall-off	18	11
Number of cases left for IHC experiments	2	9
Percentage of tissues left for IHC experiments	10	45
**Total number of PFPE tissues**	20	20
Cases of tissue fall-off after D or R steps	3	1
Cases of tissue fall-off after AR	5	3
Total cases of tissue fall-off	7	4
Number of cases left for IHC experiments	13	16
Percentage of tissues left for IHC experiments	65	80

Abbreviations: D, deparaffinization; R, rehydration; NOM, normal oral mucosa; OSCC, oral squamous cell-carcinoma; FFPE, formalin-fixed paraffin embedded; AR, antigen retrieval; IHC, immunohistochemistry.

**Table 5:** Assignment of FTIR bands for PFPE and FFPE tissues.

	Band Positions (cm^−1^)					
Vibration	PFPE NOM	FFPE NOM	PFPE OSCC	FFPE OSCC	Assignment [12] (Malek et al. 2014; Wang and Wang 2021)	
COH deformation	1010	1021	1010	1002	Glycogen/carbohydrates	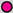
PO_2_^-^ asymmetric stretch	1085	1082	1082	1080	Nucleic acids and phospholipids	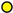
C–O stretch of the ribose ring	1113	1111	1113	1115	RNA	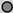
C–O stretch	1157	×	1155	×	Glycogen	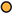
Amide III	1245	1249	1247	1245	Protein	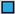
	1324	×	1326	1330	Protein	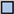
COO^-^ symmetric stretch	1419	×	1419	×	Fatty acids, amino acids	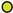
	1427	1427	1427	1431	Fatty acids, amino acids	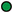
CH3 and CH2 deformations	1472	1474	1474	1472	Lipid Proteins	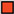
Ring CC stretch of tyrosine residues	1517	1515	1515	1515	Tyrosine Proteins	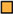
NH bend + C–N stretch Amide II	1565	1571	1571	1573	Proteins	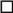
C = O + NH bend Amide I	1684	1689	1687	1684	Proteins	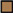

Colored shapes represent the assigned biomolecules, which can be correlated with spectra represented in Figure 5. Abbreviations: FFPE, formalin-fixed paraffin embedded; PFPE, PAXgene-fixed paraffin embedded; OSCC, oral squamous cell carcinoma; NOM, normal oral mucosa.

## Data Availability

All data pertaining to the manuscript is given in the main-text or supplementary data. Additional information can be requested from the corresponding author if and when required.

## Supplementary Materials

The following are available online at https://www.mdpi.com/article/10.3390/biom11060889/s1, Table S1: Percentage loss of tissue area from epithelium and sub-epithelium regions due to tissue tear or wrinkling, Table S2: Experimental Conditions Used for Optimization of Antigen Retrieval for Immunohistochemistry Application, Table S3. Documentation of cases of tissue section fall-off from slides during sample processing steps for immuno-histochemical staining experiments for PFPE tissue without antigen retrieval, Methods S1: Immunohistochemistry.
