# Supplementary material for: Comprehensive Evaluation of PAXgene Fixation on Oral Cancer Tissues Using Routine Histology, Immunohistochemistry, and FTIR Microspectroscopy
Source: Biomolecules. 2021 Jun 15;11(6):889. doi: 10.3390/biom11060889 (PMC8232633; doi:10.3390/biom11060889)
Supplement: Supplementary file 1 [file biomolecules-11-00889-s001.zip › biomolecules-1246684-supplementary.pdf]

Supplementary Information

Supplementary Tables

Table S1. Percentage loss of tissue area from epithelium and sub-epithelium regions due to tissue tear or wrinkling

	Epithelium		Sub-Epithelium	
	FFPE-NOM n = 13*	PFPE-NOM n = 17*	FFPE-NOM n = 13*	PFPE-NOM n = 17*
Mean Percentage Loss of Tissue Area	4.08	2.09	28.7	19.1
SEM	0.974	0.302	3.91	2.75

* Represents the number of samples excluding the samples lost due to tissue fall-off from slides

Abbreviations: FFPE, formalin-fixed paraffin embedded; PFPE; PAXgene-fixed paraffin embedded; NOM, normal oral mucosa; SEM, standard error of mean

Table S2. Experimental Conditions Used for Optimization of Antigen Retrieval for Immunohistochemistry Application

	Temperature Conditions	Instrument used	Cases of tissue fall-off/ Total number of FFPE-NOM samples	No. of samples left for IHC	Antigen Retrieved
1	65 ºC for 20 minutes<br>98 ºC for 2 minutes	Microwave	17/20	3	Yes
2	65 ºC for 30 minutes	Microwave	15/20	5	Yes
3	98 ºC for 5 minutes	Microwave	15/20	5	Partial or inconsistent retrieval
4	98 ºC for 10 minutes	Microwave	20/20	0	Not applicable
5	60 ºC for 30 minutes	Microwave	10/20	10	Partial or inconsistent retrieval
6	98 ºC for 10 minutes	Water bath	20/20	0	Not applicable
**7**	***65 ºC for 30 minutes***	***Water bath***	***4/20***	***16***	***Yes***
8	60 ºC for 30 minutes	Water bath	0/20	20	Partial or inconsistent retrieval

Abbreviations: FFPE, formalin-fixed paraffin embedded; NOM, normal oral mucosa; IHC, immunohistochemistry; ºC, degree Celsius

Table S3. Documentation of cases of tissue section fall-off from slides during sample processing steps for immuno-histochemical staining experiments for PFPE tissue *without antigen retrieval*

	Number of NOM samples	Number of OSCC samples
**Total number of PFPE tissues**	20	20
Cases of tissue fall-off after D or R steps	2	1
Total cases of tissue fall off	2	1
Number of cases left for IHC experiments	18	19
**Percentage** of tissues left for IHC experiments	**90**	**95**

Abbreviations: FFPE, formalin-fixed paraffin embedded; PFPE; PAXgene-fixed paraffin embedded; NOM, normal oral mucosa; IHC, immunohistochemistry

Supplemental Methods

Method S1. Immunohistochemistry

Stepwise laboratory protocol of immunohistochemistry with PFPE and FFPE oral buccal mucosa tissues

Material Required:

1. AUTOFROST Adhesion Microscope Slides (Cancer Diagnostics lnc. #20190710)

2. FFPE and PFPE oral buccal mucosa tissue slides.

3. Immunohistochemistry (IHC) kit (DAKO #K8023)

4. Tris buffered saline (TBS): 50 mM Tris-Cl, pH 7.5, 150 mM NaCl

5. Tris EDTA buffer pH 9(antigen retrieval buffer): 10mM Tris Base, 1mM EDTA Solution, 0.05% Tween 20, pH 9.0

6. DPX Mountant for histology (Sigma Aldrich # 44581)

Methods:

Deparaffinization

1. Bake the slides with tissue sections for 30 minutes at 65ºC incubator
2. Immerse the slides with tissue sections in xylene with two changes for 10 minutes each

Rehydration

3. Rehydrate the slides with tissue sections in decreasing gradient of alcohol.
    - 100% ethanol: 2 minutes
    - 90% ethanol: 2 minutes
    - 70% ethanol: 2 minutes

- 50% ethanol: 2 minutes
- Immerse the slides in MilliQ water for few seconds

Wash

4. Immerse the slide with tissue sections in TBS and wash 3X for 5 minutes.

Antigen Retrieval

5. Immerse the slides with tissue sections in Tris-EDTA buffer, pH 9.
6. Antigen retrieval of tissue sections was performed at 65ºC for 30 minutes in a water bath.
7. Let the slides with tissue sections cool down for 20 minutes.

Wash

8. Immerse the slide with tissue sections in TBS and wash 3X for 5 minutes.

Peroxide Block

9. Add 50-100 ul of DAKO Peroxide block from the IHC kit (ready to use) to the sections and incubate for 10 minutes.

Wash

10. Immerse the slide with tissue sections in TBS and wash 3X for 5 minutes.

Primary Antibody Incubation

11. Dilute the primary antibody in 1% BSA in TBS as per manufacturer's instructions. (p53:1:500; CK5/6, 1:500) in 1%BSA+TBS)
12. Add 50-100 of primary antibody to the tissue sections and incubate for 2.5 hours.

Wash

13. Immerse the slide with tissue sections in TBS and wash 3X for 5 minutes.

Secondary Antibody Incubation

14. Add 50-100 ul of EnVision™ FLEX /HRP (ready to use) to the tissue sections and incubate for 1 hour.

Wash

15. Immerse the slide with tissue sections in TBS and wash 3X for 5 minutes.

Chromogenic Reaction

16. MIx 10 ul of DAB chromogen in 1000ul of EnVision™ FLEX Substrate Buffer and add 50-100 ul of the mixture on the tissue sections and incubate till color appears. This step was performed under the bright-field microscope.
17. Optimization of the timing of the color reaction should be performed for each antibody used (p53; 2.5 minutes and CK5/6; 1 minute).

Wash

18. Immerse the slide with tissue sections in TBS and wash 3X for 5 minutes.

Counterstain

19. Counterstain the tissue sections with hematoxylin for 20 seconds

Wash

20. Wash the tissue sections with tap water for 2 minutes

Dehydration and Mounting

21. Dehydrate the tissue sections by immersing the slides in increasing gradient of alcohol.
    - 70% ethanol for 2 minutes
    - 100% ethanol for 2 minutes
22. Mount the slides with DPX mountant.
